# Association of Ulcerative Colitis with *FUT2* and *FUT3* Polymorphisms in Patients from Southeast China

**DOI:** 10.1371/journal.pone.0146557

**Published:** 2016-01-14

**Authors:** Dingyuan Hu, Daguan Zhang, Shuzi Zheng, Maodong Guo, Xinxin Lin, Yi Jiang

**Affiliations:** Department of Gastroenterology, The Second Affiliated Hospital of Wenzhou Medical University, Wenzhou, China; Laikon Hospital, GREECE

## Abstract

**Objectives:**

Dysbiosis of intestinal microbiota has been implicated in ulcerative colitis (UC). Fucosyltransferase (FUT) 2 and FUT3 determine expression of histo-blood group antigens in the gut and may affect the intestinal microbiota. We investigated the association between *FUT2* and *FUT3* polymorphisms and UC in Chinese patients.

**Methods:**

We genotyped *FUT2* (rs281377, rs1047781 and rs601338) and *FUT3* (rs28362459, rs3745635 and rs3894326) in 485 UC patients and 580 healthy controls using *SNaPshot*. We also evaluated expression of Lewis a and b antigens in the sigmoid colon of 7 UC patients and 7 patients with benign colonic polyps.

**Results:**

The frequencies of mutant allele (A) and genotype (GA+AA) in *FUT3* (rs3745635) were higher in UC patients than controls (*P* = 0.016, 95%*CI*: 1.339–1.699; *P* = 0.038, 95%*CI*: 1.330–1.742, respectively). Stratified analyses revealed that the frequencies of mutant allele (G) and genotype (TG+GG) of *FUT3* (rs28362459) were significantly lower in patients with extensive colitis than those with distal colitis (*P*<0.001, 95%*CI*: 0.503–0.742; *P* = 0.001, 95%*CI*: 0.567–0.786, respectively). Similar conclusions were drawn for the mutant allele (A) and genotype (GA+AA) of *FUT3* (rs3745635) in patients with extensive colitis compared to those with distal colitis (*P* = 0.006, 95%*CI*: 0.553–0.845; *P* = 0.011, 95%*CI*: 0.621–0.900, respectively). Although expression of Lewis b antigen in the sigmoid colon did not differ between UC patients and controls, Lewis a antigen expression was higher in the cryptic epithelium of both inflammatory and non-inflammatory sigmoid colon of UC patients than controls (*P* = 0.028).

**Conclusions:**

Our findings indicated that polymorphisms in *FUT3* and its intestinal expression might be associated with UC pathogenesis.

## Introduction

Inflammatory bowel diseases (IBDs) are a group of chronic and non-specific intestinal inflammatory disorders including ulcerative colitis (UC) and Crohn’s disease (CD). Although the precise etiology of IBDs is not yet fully understood, abnormal host-microbial interactions have been implicated in the pathogenesis of IBD. Mucosal and fecal bacterial analyses have suggested that patients with IBD have less complex commensal bacteria population and higher numbers of mucosal-associated bacteria than healthy individuals [1–3]. Animal models have also indicated that the composition of intestinal microbiota influenced intestinal inflammation [4]. The composition of intestinal microbiota appears to be influenced by host genetics. For instance, in patients with IBD carrying the *NOD2* and *ATG16L1* risk alleles the intestinal microbiota contains lower levels of *Faecalibacterium* and higher levels of *Escherichia* [5].

The intestinal microbiota can be influenced by Fucosyltransferase (*FUT*) *2* and *FUT3*, which control presentation of histo-blood group antigens (HBGA) on the gastrointestinal mucosa and in bodily secretions. HBGA, including ABH antigens and Lewis antigens, act not only as binding sites for intestinal microbes such as *Helicobacter pylori*, *Campylobacter jejuni*, *Norovirus* and *rotavirus* [6–8], but also a carbon source for microbes including *Escherichia coli* [9]. *FUT2* is located in chromosome 19q13, while *FUT3* is mapped to chromosome 19p13, a genomic region containing putative susceptibility loci (IBD6) for IBD [10,11]. *FUT2* influences presentation of ABH antigens in the gastrointestinal mucosa and their secretion. In people that express functional *FUT2*, termed secretors, expression of ABH antigens is widespread in the stomach and small intestine, but decreases progressively from the proximal to distal colon [12,13]. Homozygotes for loss-of-function alleles in *FUT2* lack expression of ABH antigens in the gastrointestinal mucosa and bodily secretions and account for approximately 20% of the world’s population [14–16]. *FUT2*(rs601338, G428A) is the most common polymorphism in *FUT2* Caucasian nonsecretors. In Chinese and Japanese populations, however, *FUT2* (rs601338, G428A) is rare and instead the more common missense mutation *FUT2* (rs1047781, A385T) is responsible for dramatically decreased expression of ABH antigens [15–17]. Additionally, *FUT2* (rs281377, T357C) has been identified as a common silent mutation in Chinese non-secretors [18, 19].

*FUT3* encodes α-(1,3/4)-fucosyltransferase required to synthesize Lewis a antigens, and mostly utilizes the H antigen, determined by FUT2, as a substrate to synthesize Lewis b antigen. Lewis b is mainly expressed in the proximal colon, but not expressed in the distal colon, whereas Lewis a is uniformly distributed throughout the colon [13]. Studies in Chinese populations have demonstrated that (rs28362459, T59G), (rs3745635, G508A) and (rs3894326, T1067A) were the most common polymorphic loci of *FUT3* [18]. Furthermore, mutations of *FUT3* (rs3745635) and (rs3894326) inactivate the enzyme [10, 20], while mutation of *FUT3* (rs28362459) reduces the availability of α-(1,3/4)-fucosyltransferase [10, 19].

In recent years, several studies have linked several nucleotide polymorphisms in *FUT2* to intestinal microbiota composition [21] and predisposition to CD [22, 23], celiac disease [24], type 1 diabetes [25] and primary sclerosing cholangitis [26], highlighting the essential role of host gene-microbiota interaction in autoimmune diseases. However, the conclusions drawn about the influence of *FUT2* on UC, were not consistent. Additionally, few studies have investigated the influence of *FUT3* polymorphisms on UC. In this study, we investigated the prevalence of *FUT2* and *FUT3* polymorphisms in a cohort of UC patients and healthy controls in Southeast China. We also evaluated intestinal expression of Lewis a and b antigens to further investigate the role of these genes in pathogenesis of UC.

## Materials and Methods

### Subjects

Between January 2004 and May 2015, 485 consecutive UC patients were recruited from The Second Affiliated Hospital of Wenzhou Medical University in Wenzhou city, Zhejiang province of Southeast China. UC was diagnosed according to colonoscopic, histo-pathological, radiologic and clinical findings, following the Lennard-Jones Criteria [27]. The severity of UC were evaluated by Truelove & Witt Activity Index [28]. In the corresponding period, a total of 580 age- and sex-matched healthy controls were enrolled at the Health Examination Center of The Second Affiliated Hospital of Wenzhou Medical University. Patients with autoimmune diseases, tumors, and IBD family history were excluded. Demographic data was collected (Table 1). The age and sex distribution did not differ significantly between these UC patients and controls. Of these UC patients, we obtained specimens of inflammatory lesions in the sigmoid colon during colonoscopy examination. Specimens of the adjacent non-inflammatory mucosa were also obtained in five of the 7 patients. In seven patients with benign colonic polyps, confirmed by colonoscopy examination and pathology, specimens of normal sigmoid colon mucosa were obtained during colonoscopy. The study was carried out in line with the Treaty of Helsinki and was approved by Ethics Committee of The Second Affiliated Hospital of Wenzhou Medical University. Written informed consent was obtained from all UC patients and controls.

**Table 1:** Demographic characteristics of UC patients and the controls.

Characteristics	UC	Controls	*P*
Total number	485	580	
Sex (Female/Male)	201/284	261/319	0.243
Age (years) [mean (SD)]	40.23(15.32)	41.09(17.11)	0.392
Age of onset (years) [mean (SD)]	39.05(14.47)		
Smoking [n (%)]			0.124
Current or ex-smoker	80(16.5)	117(20.2)	
Never smoked	405(83.5)	463(79.8)	
Lesion location [n (%)]			
Distal colitis	311(64.1)		
Extensive colitis	174(35.9)		
Severity of UC [n (%)]			
Mild	188(38.8)		
Intermediate	223(46.0)		
Severe	74(15.3)		
Treatment [n (%)]			
SASP/5-ASA	408(84.1)		
Prednisone	118(24.3)		
Antibiotics	170(35.1)		
Immunosuppressant	9(1.9)		
Infliximab	0(0)		
Colectomy	2(0.4)		

UC, ulcerative colitis. SD, standard deviation. SASP, sulfasalazine. 5-ASA, 5-aminosalicylic acid.

### Genomic DNA Extraction and Genotyping

Approximately 5 ml of peripheral blood was collected from each study subject in an EDTA tube. Genomic DNA was extracted from peripheral blood using the DNeasy Blood & Tissue kit (Qiagen GmbH), according to the manufacturer’s instructions. Genomic DNA was stored at 4°C for subsequent identification of genetic mutations.

We employed a Multiplex *SNaPshot* assay (Applied Biosystems, California, USA) to examine *FUT2* and *FUT3* genotypes. First, 10 ng of genomic DNA was added to a 10 μl PCR mixture containing 20 μmol dNTPs (Promega, Wisconsin, USA), 0.5 U FastStart Taq DNA polymerase (Roche, Basel, Switzerland), 1μl 10×PCR buffer with MgCl_2_ (15 mmol/L) (Roche, Basel, Switzerland) and amplification primers with a specific final concentration (Table 2). The thermal cycler conditions for multiplex PCR amplification were as follows: initial denaturation, 95°C for 5 min, amplification for 35 cycles at 94°C for 30 s, 65°C for 30 s and 72°C for 1 min, followed by a final elongation step at 72°C for 10 min. Subsequently, the PCR products were examined by electrophoresis on a 2.5% agarose gel. Second, we purified the PCR product using a mix of 1.5 U shrimp alkaline phosphatase (SAP) (New England Biolabs, Massachusetts, USA) and 2 U Exonuclease I (TAKARA, Dalian, China) at 37°C for 80 min, then 85°C for 15 min. Third, the multiplex SNaPshot sequencing reactions were carried out in a final volume of 7 μl containing 2 μl of purified multiple PCR products, 1 μl SNaPshot Multiplex Mix, 1 μl 5×Sequencing buffer (Applied Biosystems) and 3 μl *SNaPshot* sequencing primers (the final concentrations are listed in Table 2). The thermal cycler conditions were: initial denaturation at 96°C for 1 min followed by 25 cycles at 96°C for 10 s, 52°C for 5 s and 60°C for 30 s. Then depuration of product was performed with 1 U SAP at 37°C for 60 min and 75°C for 15 min. Finally, 1.5 μl of *SNaPshot* products were genotyped using the ABI 3730 Genetic Analyzer before they were mixed with 8 μl HiDi^TM^ formamide and 0.5 μl GeneScan-120LIZ size standard (Applied Biosystems). Data were analyzed using GeneMapper 4.0 (Applied Biosystems). In order to guarantee quality of the study, about 3 percent of the samples were randomly selected and re-genotyped by direct sequencing. The regenotyping and *SNaPshot* results were in complete accordance with original results.

**Table 2:** Amplification and extension primers of *FUT2* and *FUT3*.

SNP	Amplification Primer (5′→3′)	Size (bp)	Conc.(μM)	Extension primer (5′→3′)	Size (bp)	Conc. (μM)
*FUT2* rs281377	F:TCAACATCAAAGGCACTGGGACC R:TGGCGGAGGTGGTGGTAGAA	338	2.5	**18T**-TGGCAGAACTACCACCTGAA	38	1
*FUT2* rs1047781	F:TCAACATCAAAGGCACTGGGACC R:TGGCGGAGGTGGTGGTAGAA	338	2.5	**38T**-TGGAGGAGGAATACCGCCAC	58	1
*FUT2* rs601338	F:TCAACATCAAAGGCACTGGGACC R:TGGCGGAGGTGGTGGTAGAA	338	2.5	CACCGGCTACCCCTGCTCCT	20	1
*FUT3* rs28362459	F:AAGAAACACACAGCCACCAGCAG R:AATGACCCTCACTCCTCTCTCCTCT	137	1	**31T**-GCGCCGCTGTCTGGCCGCAC	51	1
*FUT3* rs3745635	F:GGCTGAGTCCGGCTTCCAGTT R:TATCATGTCCAACCCTAAGTCACGC	280	1	**5T**-CCGACATCTTCACGCCCTAC	25	1
*FUT3* rs3894326	F:AGGTCCCTAGCAGGCAAGTCTTC R:CTGGGCACTGGATTTCTGCAAGG	254	1	**21T**-CAGGTACCAGACGGTGCGCAGCA	44	1

Bold characters refer to the 5’ poly-thymidine tail.

### Immunohistochemistry

For immunohistochemical analysis, formalin-fixed paraffin-embedded tissue sections were de-paraffinized with xylene and rehydrated with ethanol solutions and distilled water. Antigen retrieval was performed by heating the sections in EDTA antigen retrieval buffer (pH 9.0) at 99°C for 25 min. Then the slides were washed in PBS (pH 7.4) three times for 5 min. After blocking in endogenous peroxidase with 3% hydrogen peroxide solution at room temperature for 25 min, the slides were again washed in PBS (pH 7.4) three times for 5 min. To block unspecific antibody binding, sections were incubated with 3% BSA (Solarbio, Beijing, China) for 30 min. Sections were then incubated overnight at 4°C in PBS (pH 7.4) containing primary antibodies, blood group Lewis a and b antibodies (Catalog number: sc-51512 and sc-51513, Santa Cruz Biotech, Texas, U.S.A.) at 1:20 in PBS (pH 7.4). Sections were washed three times in PBS then incubated with polyclonal goat anti-mouse immunoglobulins/HRP (Dako, Glostrup, Denmark) at room temperature for 50 min. The the slides were rinsed in PBS three times for 5 min and HRP was added. Rabbit/Mouse (DAB+). Harris staining was performed to highlight the nucleus of cells.

Lewis antigen expression was evaluated quantitatively using the Image Pro Plus 6.0 analysis system (Media Cybernetics, Silver Spring, MD) by calculating the mean density, which was integrated optical density (IOD) divided by area of interest [29]. Three 200X fields under microscope of each slide were randomly selected and the mean density was obtained for further statistic analysis.

### Statistical Analysis

Hardy-Weinberg equilibrium for genotypes was evaluated by *chi-square* test both in UC patients and the controls. Either *chi-square* or *Fisher’s* exact test was applied to compare categorical variables such as alleles and genotypes. Unconditional logistic regression analysis was employed to investigate the allelic and genotypic distributions in UC patients, stratified by their clinical characteristics. Covariants included age, gender, smoking, lesion location and severity of UC. Linkage disequilibrium (LD) was estimated and visualized by *Haploview* 4.2. Haplotypes were reconstructed and haplotype frequencies were calculated by *Phase* 2.1 software [30]. Mann-Whitney U-test was applied to compare expression of Lewis antigens. All statistical data were input into *SPSS* 17.0 software (SPSS for Windows version 17.0, Chicago, IL, USA). A two-tailed *P* value less than 0.05 was considered significant.

## Results

### Comparison of alleles and genotypes of *FUT2* and *FUT3* between UC patients and controls

In this study, the genotype distributions of *FUT2* and *FUT3* were shown to be in good agreement with Hardy-Weinberg equilibrium both in UC patients and the controls (all *P*>0.05). The allele and genotype frequencies of SNPs in *FUT2* did not differ significantly between UC patients and the controls (all *P*>0.05). However, the frequencies of mutant allele (A) and genotype (GA+AA) in *FUT3* (rs3745635) were higher in UC patients than the controls (*P* = 0.016, 95%*CI*: 1.339–1.699; *P* = 0.038, 95%*CI*: 1.330–1.742, respectively). In addition, the most common homozygous mutation of *FUT2* (rs601338), which led to nonsecretors in Caucasians, was not observed in our study. Several healthy controls (17.3%) were homozygous for *FUT2* (rs1047781 T), thus nonsecretors (Table 3, Dataset in S1 Dataset).

**Table 3:** Allelic and genotypic distributions of *FUT2* and *FUT3* between UC patients and the controls.

Genotype/allele	Controls(580) n(%)	UC (485)n(%)	OR (95%CI)	*P*
*FUT2* rs281377				
TT	441(76.0)	356(73.4)		
TC	126(21.7)	114(23.5)		
CC	13(2.2)	15(3.1)		
TC+CC	139(24.0)	129(26.6)	0.871(1.150–1.517)	0.324
allele C	152(13.1)	144(14.8)	0.904(1.156–1.478)	0.247
*FUT2* rs1047781				
AA	160(27.6)	155(32.0)		
AT	314(54.1)	247(50.9)		
TT	106(18.3)	83(17.1)		
AT+TT	420(72.4)	330(68.0)	0.623(0.811–1.056)	0.119
allele T	526(45.3)	413(42.6)	0.753(0.894–1.061)	0.200
*FUT2* rs601338				
GG	575(99.1)	479(98.8)		
GA	5(0.9)	6(1.2)		
AA	0(0)	0(0)		
GA+AA	5(0.9)	6(1.2)	0.437(1.441–4.749)	0.547
allele A	5(0.4)	6(0.6)	0.437(1.438–4.726)	0.548
*FUT3* rs28362459				
TT	332(57.2)	281(57.9)		
TG	219(37.8)	174(35.9)		
GG	29(5.0)	30(6.2)		
TG+GG	248(42.8)	204(42.1)	0.761(0.972–1.241)	0.819
allele G	277(23.9)	234(24.1)	0.830(1.013–1.237)	0.895
*FUT3* rs3745635				
GG	435(75.0)	336(69.3)		
GA	136(23.4)	133(27.4)		
AA	9(1.6)	16(3.3)		
GA+AA	145(25.0)	149(30.7)	1.016(1.330–1.742)	0.038
allele A	154(13.3)	165(17.0)	1.055(1.339–1.699)	0.016
*FUT3* rs3894326				
TT	471(81.2)	406(83.7)		
TA	105(18.1)	76(15.7)		
AA	4(0.7)	3(0.6)		
TA+AA	109(18.8)	79(16.3)	0.611(0.841–1.156)	0.286
allele A	113(9.7)	82(8.5)	0.635(0.856–1.153)	0.305

### Haplotype analysis of *FUT2* and *FUT3*

**Fig 1:**
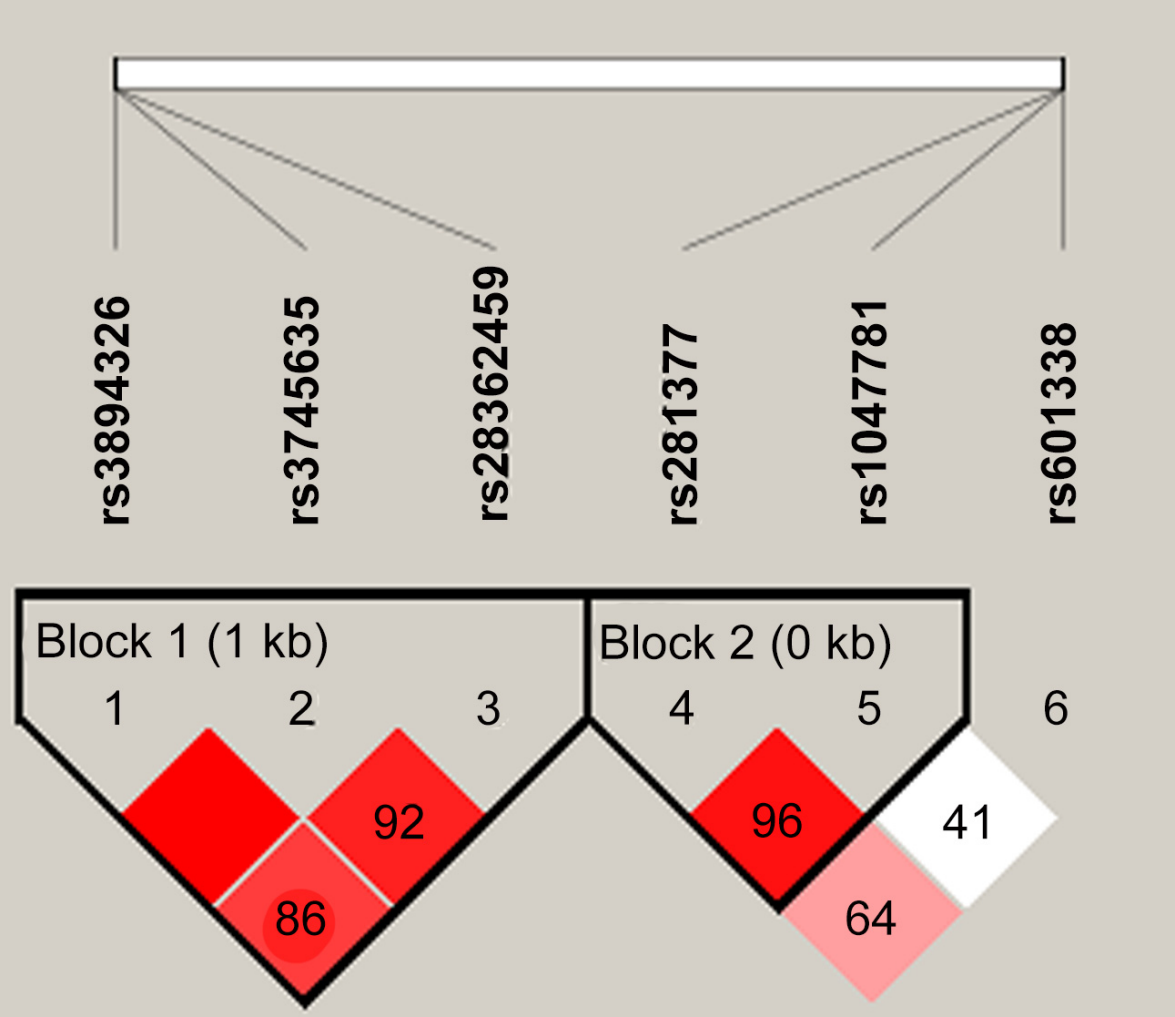
Linkage disequilibrium patterns of *FUT2* and *FUT3* in Chinese Han populations. Numbers indicated the percentage of D' between 2 SNPs and in the dark area which have no digital represents D' = 1. Dark color indicated strong connection. Block 1 illustrated linkage disequilibrium in the 3 SNP in *FUT3*, and block 2 showed that rs281377 was in linkage disequilibrium with rs1047781 in *FUT2*.

Two LD blocks were presented as follows: block rs281377-rs1047781 in *FUT2* (*D*' = 0.96, r^2^ = 0.11) and block rs3894326-rs3745635-rs28362459 in *FUT3* [rs3894326/rs3745635 (*D'* = 1, r^2^ = 0.01), rs3894326/rs28362459 (*D*' = 0.86, r^2^ = 0.23), rs3745635/rs28362459 (*D'* = 0.92, r^2^ = 0.47)] (Fig 1). Furthermore, we conducted a haplotype analysis in all study subjects to investigate whether any specific haplotype would confer risk of or protection from UC. No haplotypes differed significantly between UC patients and the controls (all *P*>0.05, Table 4).

**Table 4:** Haplotype frequencies of *FUT2* and *FUT3* in patients with UC and the controls.

Groups	*FUT2* (rs281377-rs1047781)			*FUT3* (rs3894326- rs3745635-rs28362459)		
	TT	TA	CA	TGT	TAG	AGG
UC	0.425	0.427	0.148	0.733	0.158	0.071
Controls	0.450	0.419	0.127	0.752	0.129	0.093

No positive results were obtained for each haplotype of *FUT2* and *FUT3* between UC patients and the controls (all *P* >0.05). The haplotype frequencies lower than 0.030 were not presented in the table.

### Association between *FUT2* and *FUT3* polymorphisms and clinical characteristics of patients with UC

Unconditional logistic regression analysis was used to evaluate the associations between *FUT2* and *FUT3* polymorphisms and clinical characteristics of UC patients. Covariants included age, gender, smoking, lesion location and severity of UC. The frequencies of mutant allele (G) and genotype (TG+GG) of *FUT3* (rs28362459) were significantly lower in patients with extensive colitis than in those with distal colitis (*P*<0.001, 95%*CI*: 0.503–0.742; *P* = 0.001, 95%*CI*: 0.567–0.786, respectively). The frequencies of mutant allele (A) and genotype (GA+AA) of *FUT3* (rs3745635) in patients with extensive colitis were significantly lower than in patients with distal colitis (*P* = 0.006, 95%*CI*: 0.553–0.845; *P* = 0.011, 95%*CI*: 0.621–0.900, respectively) (Table 5). However, none of the six SNPs of *FUT2* and *FUT3* were significantly associated with UC severity in this sample (all *P*>0.05).

**Table 5:** Association of *FUT2* and *FUT3* polymorphisms with clinical characteristics of UC patients.

Genotype/Allele	Distal colitis(n = 311)(%)	Extensive colitis(n = 174)(%)	OR(95%CI)	*P*
*FUT2* rs281377				
TT	231(74.3)	125(71.8)		
TC+CC	80(25.7)	49(28.2)	0.746(1.132–1.717)	0.560
T allele	532(85.5)	294(84.5)		
C allele	90(14.5)	54(15.5)	0.753(1.086–1.566)	0.660
*FUT2* rs1047781				
AA	90(28.9)	65(37.4)		
AT+TT	221(71.1)	109(62.6)	0.461(0.683–1.012)	0.057
A allele	346(55.6)	211(60.6)		
T allele	276(44.4)	137(39.4)	0.623(0.814–1.063)	0.131
*FUT2* rs601338				
GG	308(99.0)	171(98.3)		
GA+AA	3(1.0)	3(1.7)	0.360(1.801–9.022)	0.468
G allele	619(99.5)	345(99.1)		
A allele	3(0.5)	3(0.9)	0.360(1.794–8.938)	0.469
*FUT3* rs28362459				
TT	162(52.1)	119(68.4)		
TG+GG	149(47.9)	55(31.6)	0.340(0.503–0.742)	0.000
T allele	450(72.3)	286(82.2)		
G allele	172(27.7)	62(17.8)	0.409(0.567–0.786)	0.001
*FUT3* rs3745635				
GG	202(65.0)	134(77.0)		
GA+AA	109(35.0)	40(23.0)	0.362(0.553–0.845)	0.006
G allele	502(80.7)	303(87.1)		
A allele	120(19.3)	45(12.9)	0.429(0.621–0.900)	0.011
*FUT3* rs3894326				
TT	253(81.4)	153(87.9)		
TA+AA	58(18.6)	21(12.1)	0.350(0.599–1.025)	0.060
T allele	562(90.4)	326(93.7)		
A allele	60(9.6)	22(6.3)	0.381(0.632–1.050)	0.074

The covariants also included sex, age of onset, smoking, severity of the disease. Nevertheless, these covariants did not affect the allelic and genotypic distributions of *FUT2* and *FUT3* in UC patients and the controls.

### Expression of Lewis a and b antigens in the sigmoid colon

**Fig 2:**
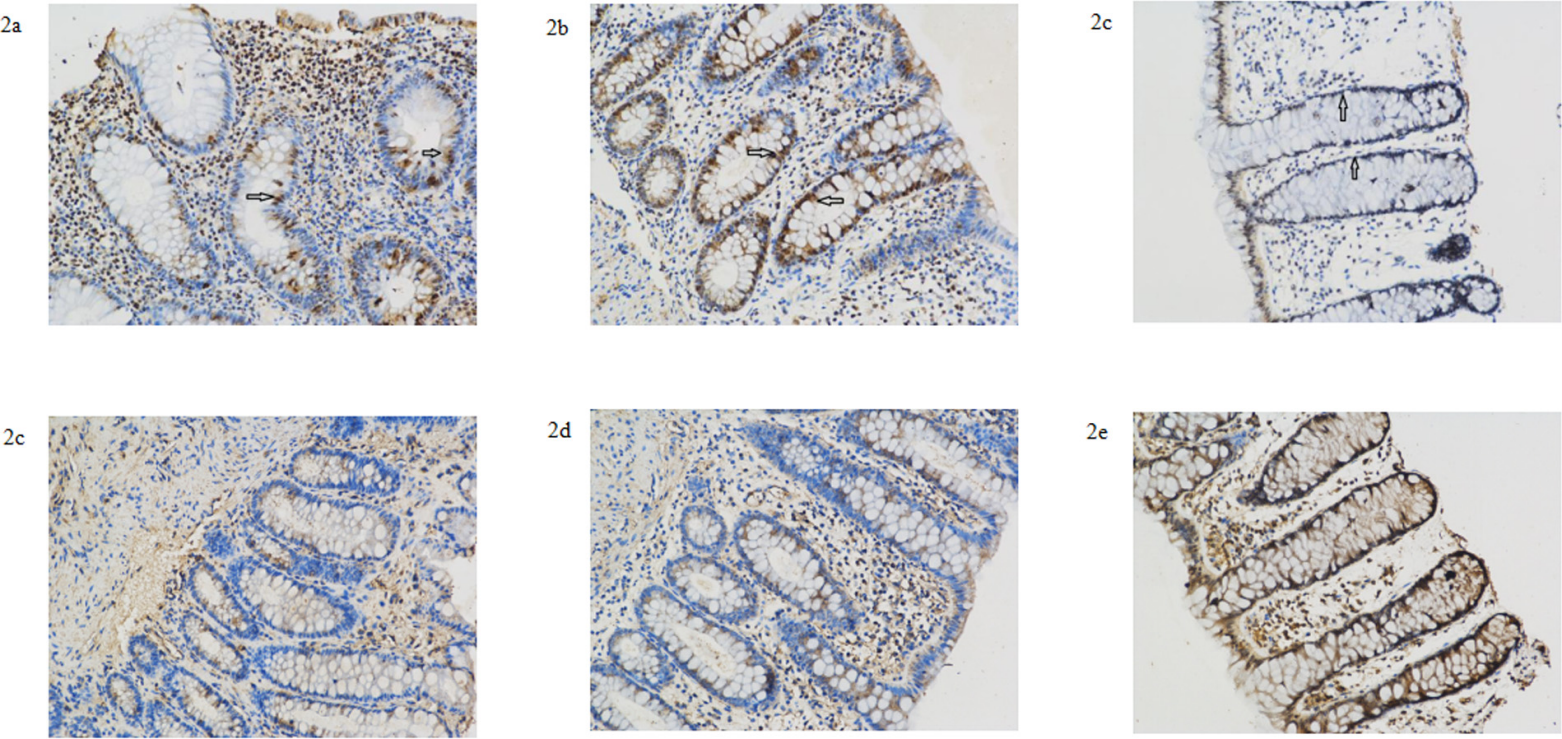
Representative Sigmoid Colon specimens of UC patients and patients with benign colonic polyps. Expression of Lewis a antigen (a-c) and Lewis b antigen (d-f) in the sigmiod specimens of inflammatory and adjacent non-inflammatory tissue of UC patients and normal controls. Samples a, b, d, e were derived from patient 5, described in Table 6. Samples c and f were derived from normal tissue of patients with benign colonic polyps. Immunohistochemical staining indicated increased expression of Lewis a antigen in the cryptic epithelium in inflammatory lesions from UC patients and normal mucosa from patients with benign colonic polyps (see arrows in a-c). Expression in the epithelium did not differ dramatically between UC patients and patients with benign colonic polyps. Expression of Lewis b antigen did not differ dramatically between the three groups (d–f).

We used immunohistochemical staining techniques to investigate expression of Lewis a and b antigens in the sigmoid colon of 7 UC patients and 7 patients with benign colonic polyps. Lewis a and b were expressed in the sigmoid colon of all studied samples, regardless of the *FUT2* or *FUT3* genotype (Fig 2, Table 6). In the normal mucosa of patients with benign colonic polyps, Lewis a was mostly expressed in the epithelium, while expression of Lewis b was observed in the cells and extracellular matrix. Immunohistochemical staining of Lewis a indicated increased expression in the cryptic epithelium of inflammatory lesions in UC patients than the normal mucosa of patients with benign colonic polyps (*P* = 0.028), although there was no difference of Lewis a expression in surface epithelium between the two groups. Notably, the extent of Lewis a staining did not differ significantly between inflammatory lesions and adjacent non-inflammatory mucosa of UC patients. In addition, the expression of Lewis b did not differ significantly among the inflammatory lesions, adjacent non-inflammatory mucosa of UC patients and normal control mucosa.

**Table 6:** Demographic characteristics of UC patients for immunohistochemistry study.

Case	Sex	Age	Smo-king	Lesion location	Disease severity	FUT2 Genotype	FUT3 Genotype
1	Male	56	yes	Distal	Mild	FUT2 rs1047781 ATFUT2 rs281377 CT	FUT3 rs28362459 GTFUT3 rs3894326 AT
2	Female	45	no	Distal	Severe	FUT2 rs1047781 TT	Wild type
3	Female	22	no	Extensive	Mild	Wild type	FUT3 rs28362459 GT
4	Male	42	no	Distal	Mild	Wild type	Wild type
5	Male	47	yes	Distal	Intermediate	Wild type	Wild type
6	Female	42	no	Distal	Intermediate	Wild type	Wild type
7	Female	51	no	Extensive	Intermediate	Wild type	Wild type

One UC patient who carried homozygous mutation of *FUT2* (rs1047781) expressed same levels of Lewis a and b antigens. This might indicate that *FUT2* genotypes have a minimal effect on expression of Lewis a and b antigens. We also noticed that expression of Lewis a and b antigen was decreased in one UC patient carrying mutant genotypes of *FUT3* (rs28362459 GT, rs3894326 AT) and *FUT2* (1047781 AT, rs281377 CT).

## Discussion

In this study, we investigated the frequency of mutations in *FUT2* and *FUT3* in Chinese patients with UC and healthy controls. In this population we found that *FUT2* polymorphisms were not significantly associated with UC. However, the frequencies of mutant allele (A) and genotype (GA+AA) of *FUT3* (rs3745635) were higher in UC patients than in the controls. Furthermore, stratified analyses also indicated that the mutant alleles and genotypes of *FUT3* (rs28362459 and rs3745635) were more common in patients with distal colitis than those with extensive colitis. These findings suggested that mutations in *FUT3* not only are associated with UC, but also might affect the location of lesions.

To our knowledge, the role of FUT3 in UC has not previously been reported. However, recent studies have linked several mutations in *FUT3* to the risk of colon cancer [31], peptic ulcer, atrophic gastritis [32,33], Norovirus infection [34], type 2 diabetes [35] and coronary heart disease [36]. Moreover, the products of *FUT3*, Lewis a and b antigen, are involved in host-microbial interactions [6–9].

Next, we used immunohistochemistry to evaluate expression of Lewis a and b antigens in the sigmoid colon of UC patients and patients with benign colonic polyps. In our study, Lewis a and b were expressed in the sigmoid colon of all studied samples, including one UC patient (case 2) who carried homozygous mutation of FUT2 rs1047781. This might indicate that the FUT2 genotypes have a minimal effect on expression of Lewis a and b antigens.

Notably, expression of Lewis a and b antigen remained unchanged in one UC patient (case 3) with mutant *FUT3* (rs28362459) genotype GT, while decreased in one UC patient (case 1) carrying mutant genotypes of *FUT3* (rs28362459 GT, rs3894326 AT) and *FUT2* (1047781 AT, rs281377 CT). These observations suggested that heterozygous mutation of rs3745635 reduced the function of α-(1,3/4)-fucosyltransferase more significantly than rs28362459. These observations may suggest a cumulative effect of *FUT3* mutations on its expression.

In comparison to healthy control samples, expression of Lewis a was higher in the cryptic epithelium in both inflammatory lesions and non-inflammatory tissue from UC patients, although no difference was found in surface epithelium between these groups. This finding suggested the potential role of Lewis a antigen in the pathogenesis of UC. Notably, the finding that expression of Lewis a antigen was also increased in non-inflammatory tissue might indicate that Lewis a antigen expression was upregulated before inflammation occurred. However, the sample size involved in the genotype-phenotype study is too small to draw any precise conclusions. Studies involving a larger number of patients and genotypes will be required to illustrate the genotype-phenotype effect of the two genes.

Lewis antigens have previously been implicated in intestinal microbiota composition [6, 21, 34]. The intestinal microbiota of individuals with negative Lewis blood groups were recently reported to contain a less rich and diverse range of bacteria than those with Lewis a phenotype [21]. In addition, fucose released from Lewis antigens was reported to play a key role in the pathogenicity and metabolism of enterohaemorrhagic *Escherichia coli* (EHEC) [9].

Some studies have previously explored the association between *FUT2* polymorphisms and IBD. McGovern *et al*. found that in a Caucasian population non-secretion, caused by a *FUT2* (rs601338) polymorphism, was associated with CD, but not UC. However, in a Finnish population the wild GG genotype of *FUT2* (rs601338) was associated with enhanced risk of UC [24]. And in a Chinese Han population mutations in *FUT2* (rs281377 and rs601338) predisposed patients to UC, while *FUT2* (rs1047781 and rs601338) polymorphisms were associated with UC in Uyghur population from northwest China [37]. In this study we found no association between *FUT2* polymorphisms and UC. Although our finding may indicate the absence of association between UC and *FUT2* polymorphisms in Chinese population, the conclusions are limited by the relatively small sample size analyzed in this study. Given the multifactorial nature of UC, many rare genetic variants may contribute to its development, and a wide range of rare variants, or the potential dependency of multiple variants, is not easily identified in these relatively small sample sizes.

Our results, however, suggested that mutations in *FUT3* and its intestinal expression might be associated with UC susceptibility in Chinese patients. Studies involving larger cohort of UC patients will be required to validate the role of *FUT3* in UC.

## Supporting Information

S1 Dataset Individual genotype distributions of FUT2 and FUT3 in UC patients and the controls. (XLSX)
